# Supplementary material for: Recognition and cleavage of human tRNA methyltransferase TRMT1 by the SARS-CoV-2 main protease
Source: eLife. 2025 Jan 7;12:RP91168. doi: 10.7554/eLife.91168 (PMC11706605; doi:10.7554/eLife.91168)
Supplement: Figure 2—source data 1. [file elife-91168-fig2-data1.docx]

| **TRMT1 Co-Incubation** | ***k_obs_***  **(hr^-1^)** | ***k_obs_***  **+/-** |  | ***K_D_***  **(µM)** | ***K_D_***  **+/-** |
| --- | --- | --- | --- | --- | --- |
| M^pro^ WT | n.d. | n.d. |  | 4.76 | 1.08 |
| M^pro^ C145A | 1.97 | 0.07 |  | 0.86 | 0.03 |
| No Protease | 2.10 | 0.05 |  | 0.80 | 0.05 |

**Figure 2–source data 1.** Table of methyltransferase activity (*k*_obs_) and tRNA binding affinity (*K*_D_) parameters for TRMT1 after an 18-hour incubation with M^pro^ WT, C145A, or no protease, corresponding to the fits of plots presented in **Figure 2C** and **2D**, respectively. TRMT1 tRNA modifying activity was measured by radiolabel-based methyltransferase assays with *S*-[methyl-^14^C]-adenosyl methionine and fit to a first-order exponential to obtain *k*_obs_ (see also **Figure 2C**); TRMT1 incubated with M^pro^ WT did not have activity during the 4-hour time course, so *k*_obs_ is listed as n.d. (not determined) for this condition. TRMT1-tRNA binding affinity was determined by EMSA experiments (see **Figure 2–figure supplement 1**) and fit to a standard single-site ligand binding equation to obtain *K_D_*s (see also **Figure 2D**). All kinetic and binding experiments were carried out in triplicate and errors are reported above as the standard error of the fits shown **Figure 2C** and **2D**.
