# Supplementary figures and images for: Recognition and cleavage of human tRNA methyltransferase TRMT1 by the SARS-CoV-2 main protease
Source: eLife. 2025 Jan 7;12:RP91168. doi: 10.7554/eLife.91168 (PMC11706605; doi:10.7554/eLife.91168)

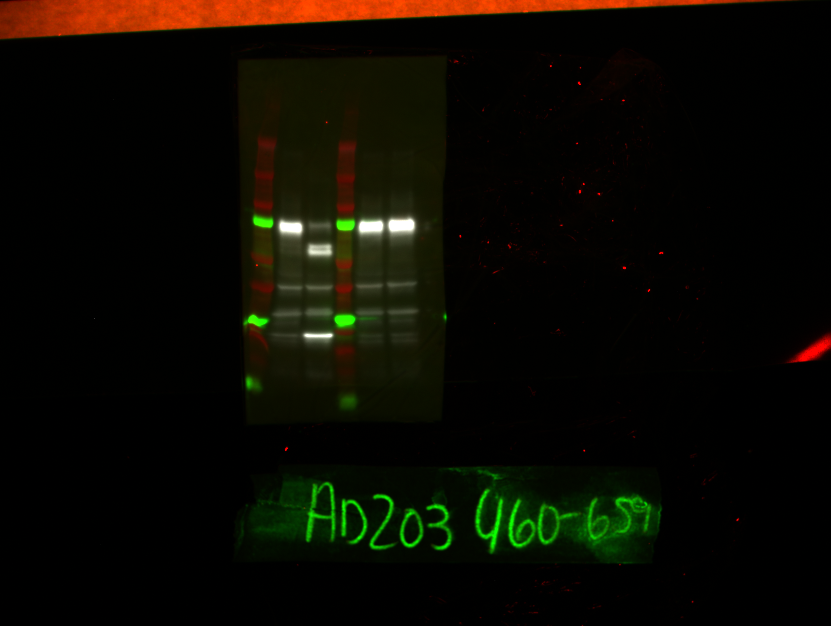

Supplement: Figure 2—source data 2. [file elife-91168-fig2-data2.zip › Figure 2A_460-659.png]

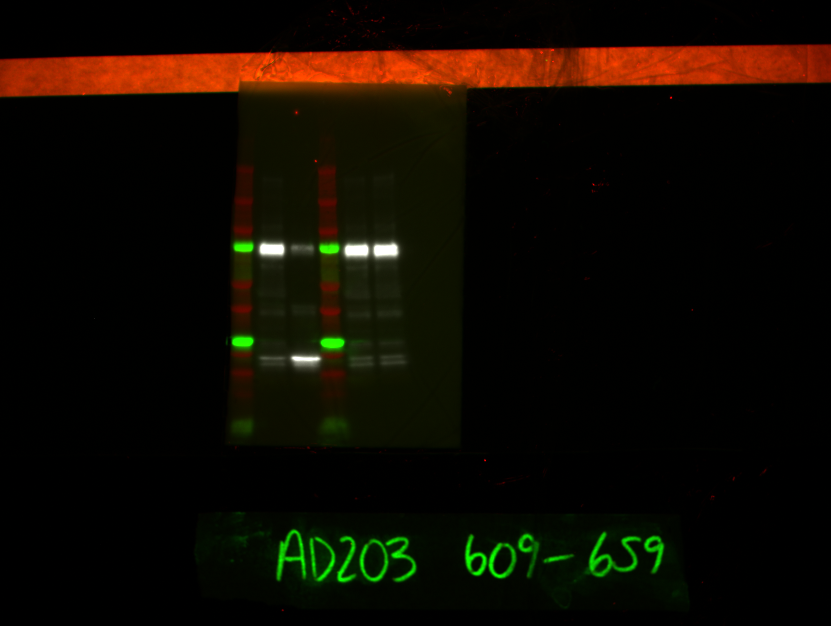

Supplement: Figure 2—source data 2. [file elife-91168-fig2-data2.zip › Figure 2A_609-659.png]

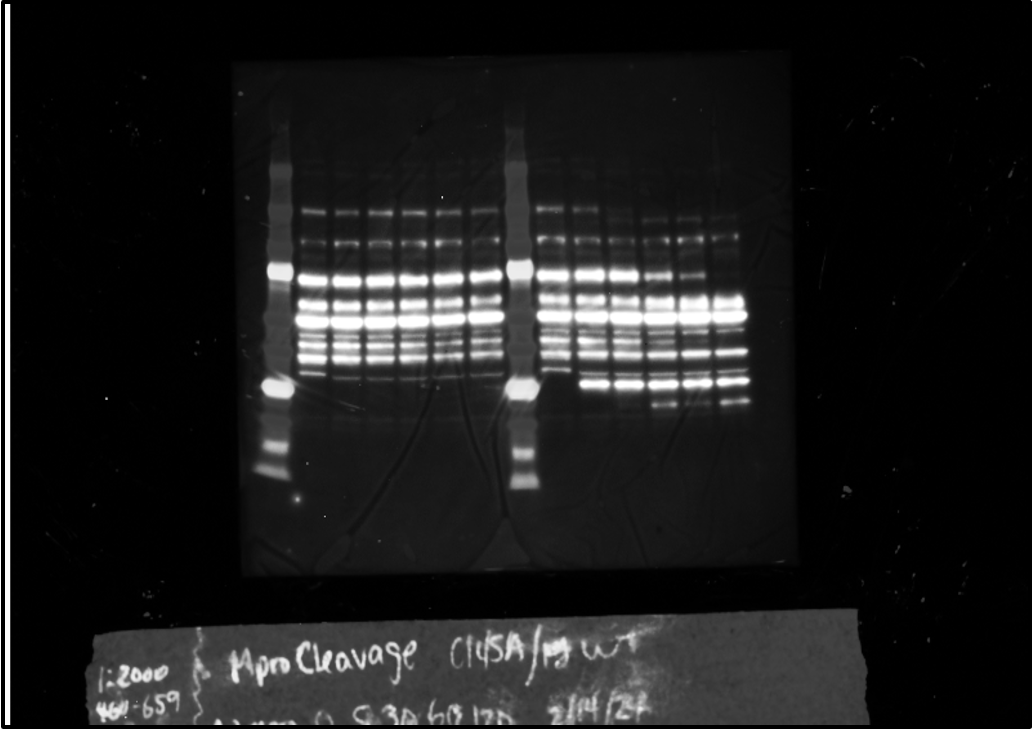

Supplement: Figure 2—source data 2. [file elife-91168-fig2-data2.zip › Figure 2B_460-659.png]

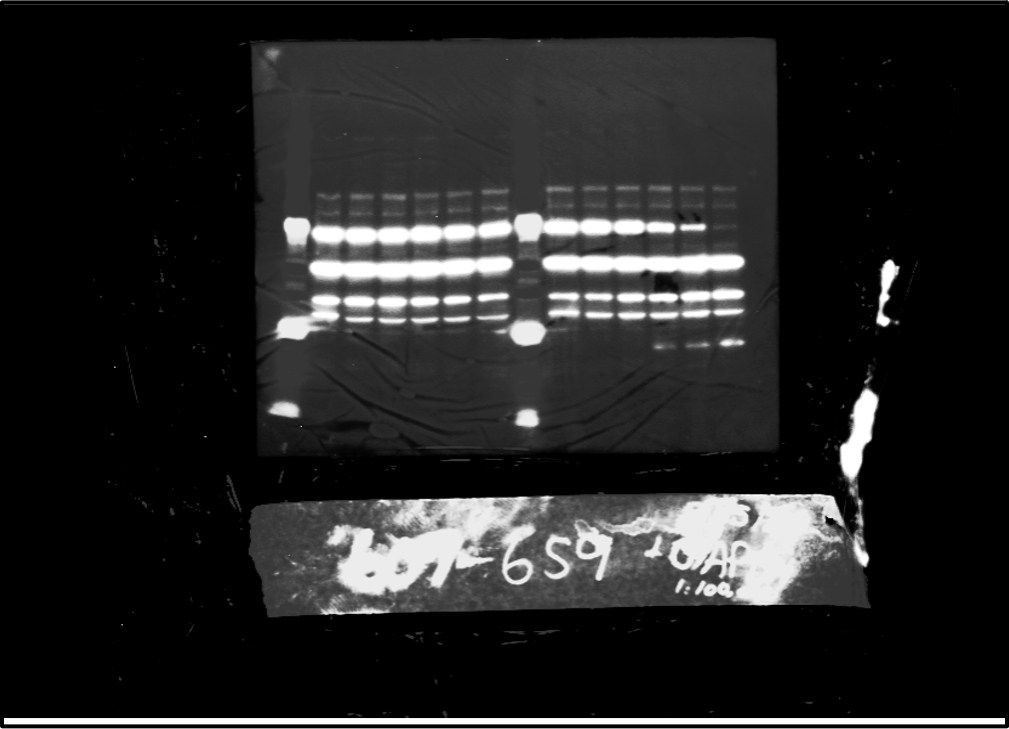

Supplement: Figure 2—source data 2. [file elife-91168-fig2-data2.zip › Figure 2B_609-659.png]

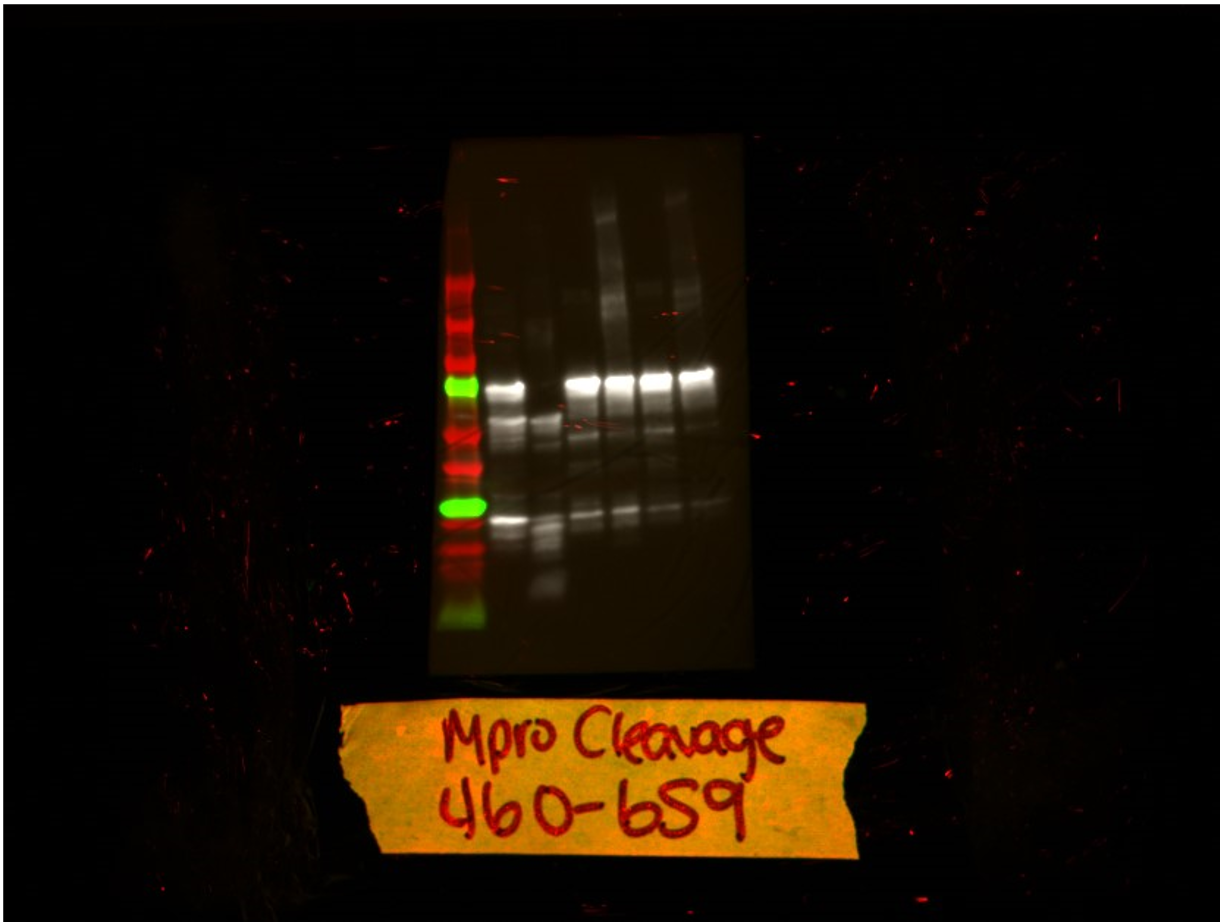

Supplement: Figure 2—source data 2. [file elife-91168-fig2-data2.zip › Figure 2C.png]

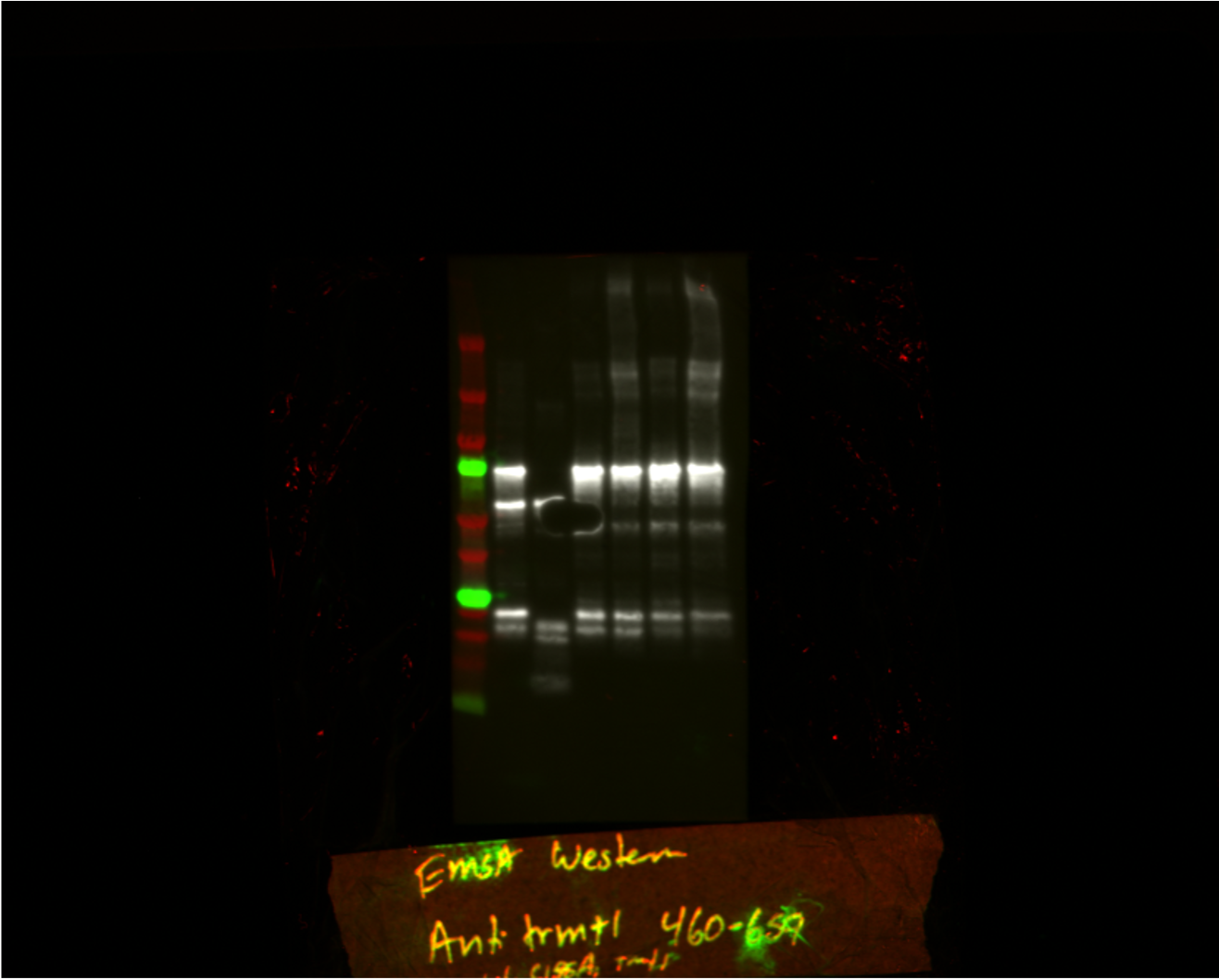

Supplement: Figure 2—source data 2. [file elife-91168-fig2-data2.zip › Figure 2D.png]

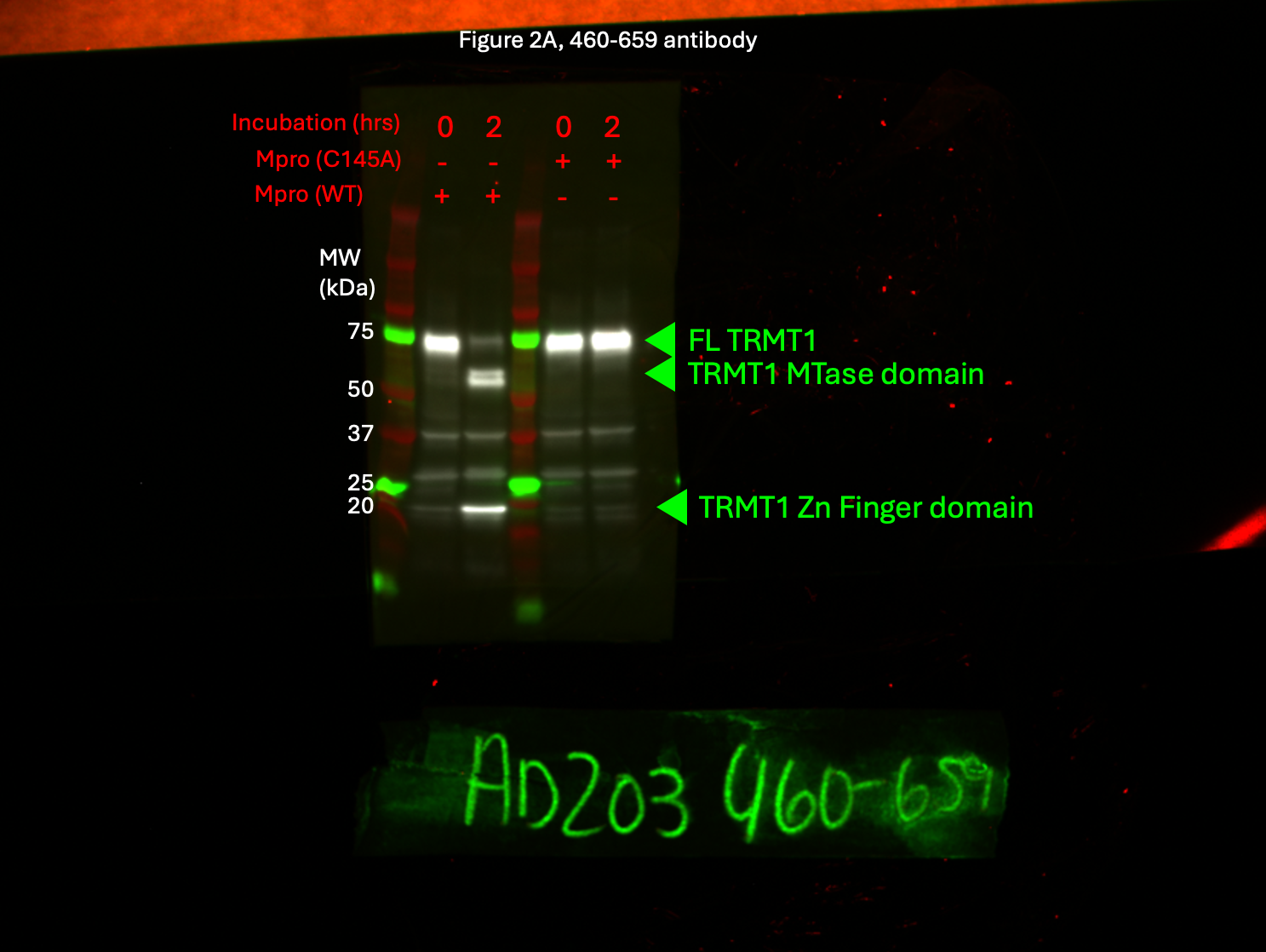

Supplement: Figure 2—source data 3. [file elife-91168-fig2-data3.zip › Figure 2A-460-659-annotated-gel.png]

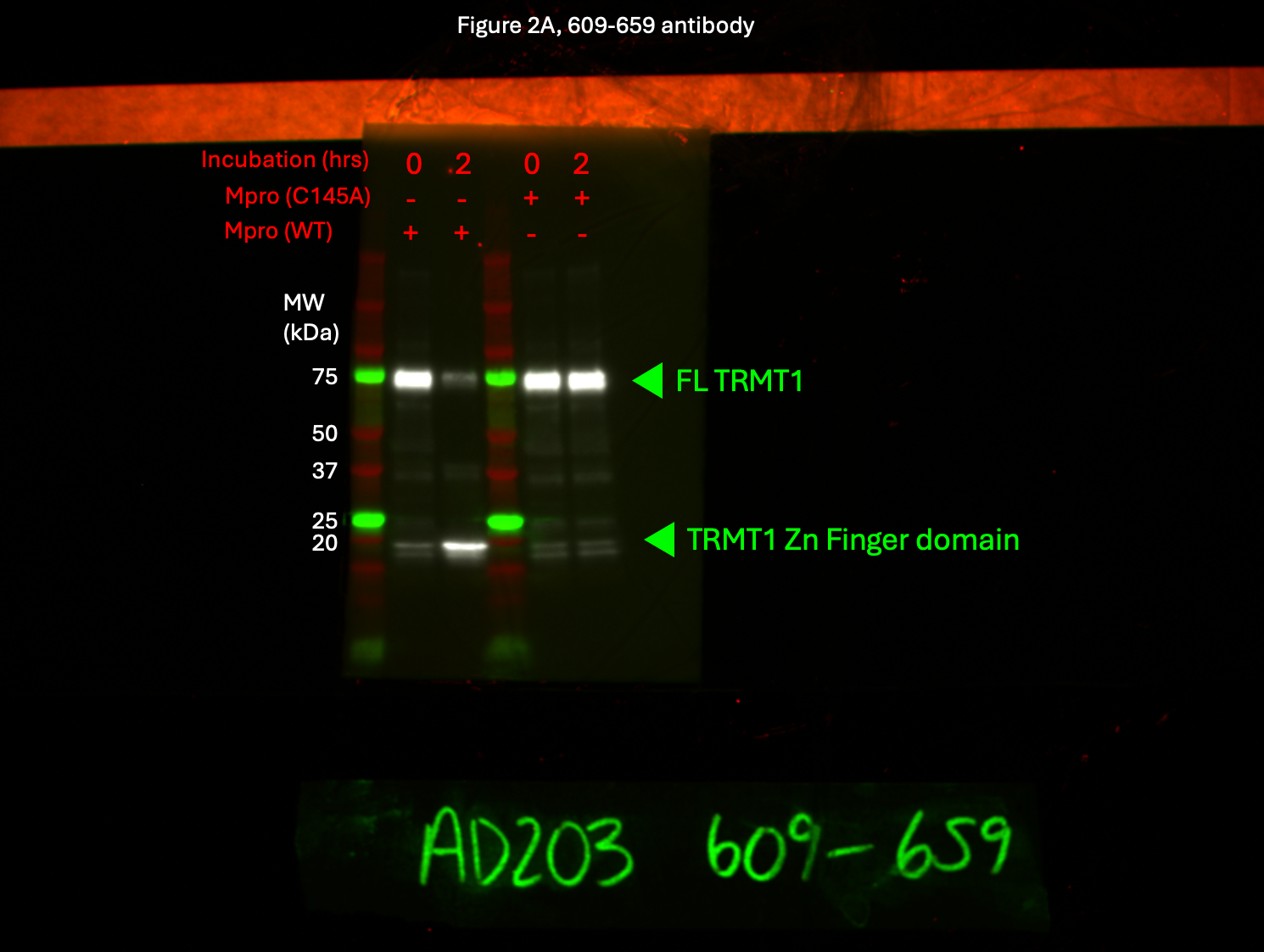

Supplement: Figure 2—source data 3. [file elife-91168-fig2-data3.zip › Figure 2A-609-659-annotated-gel.png]

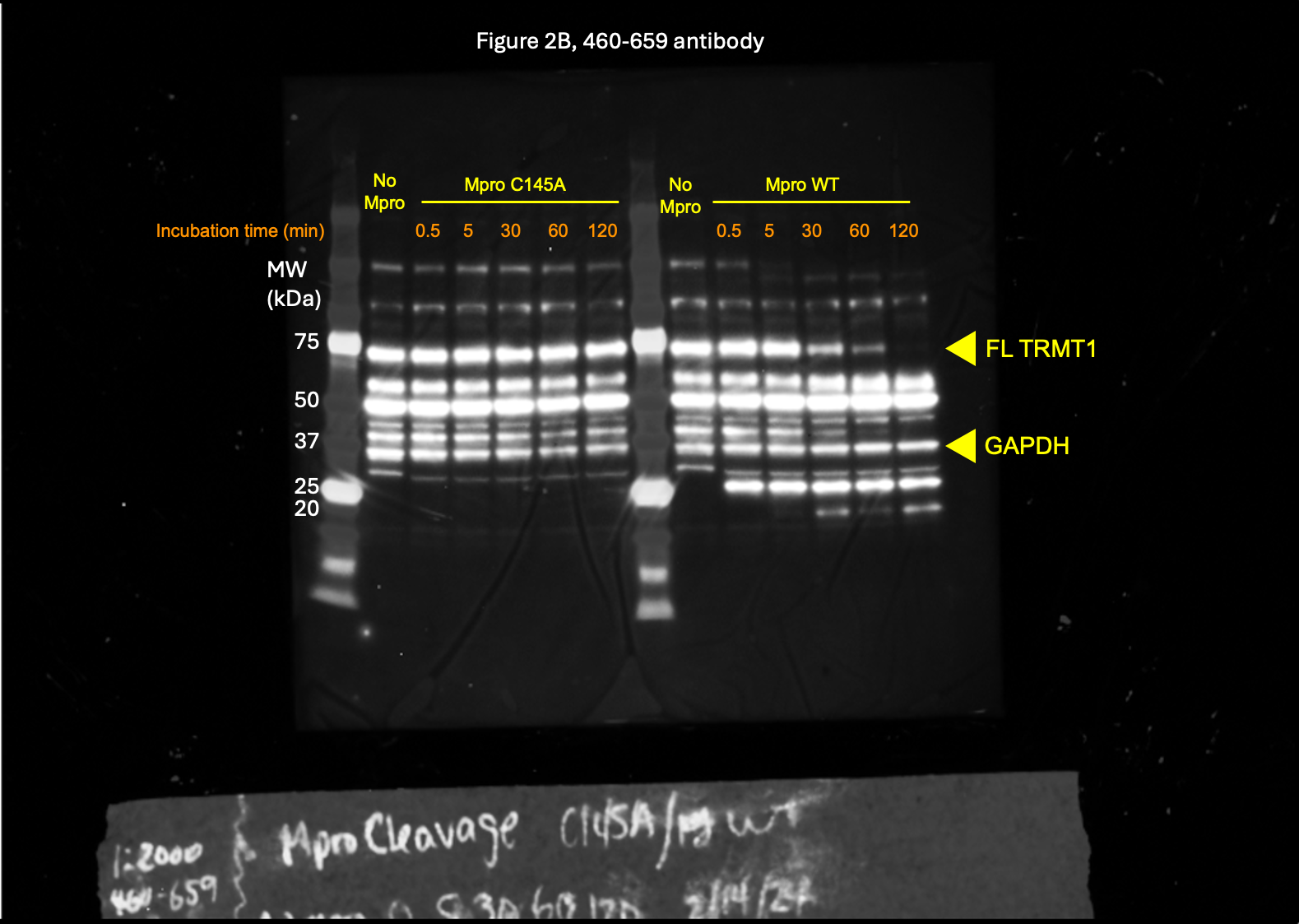

Supplement: Figure 2—source data 3. [file elife-91168-fig2-data3.zip › Figure 2B-460-659-annotated-gel.png]

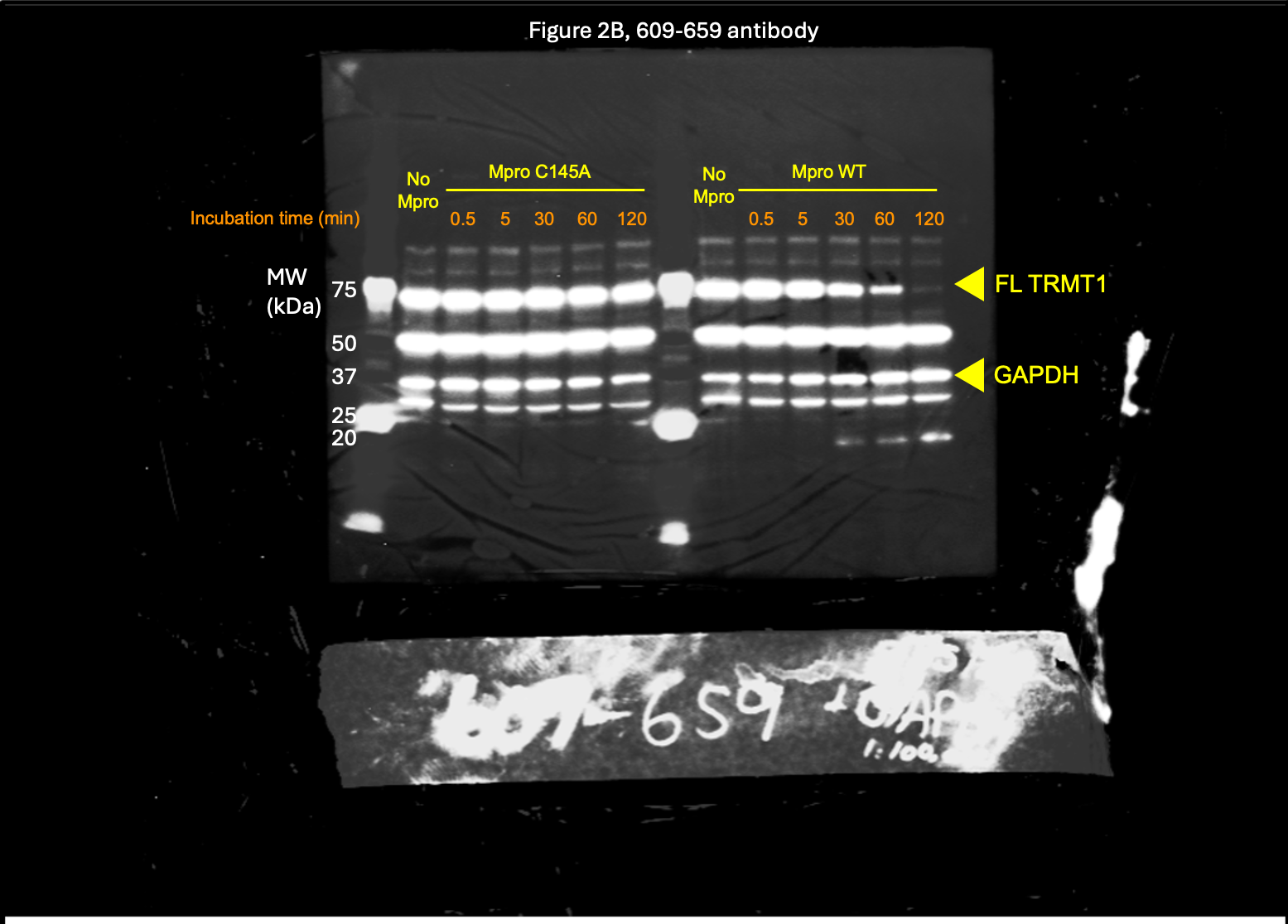

Supplement: Figure 2—source data 3. [file elife-91168-fig2-data3.zip › Figure 2B-609-659-annotated-gel.png]

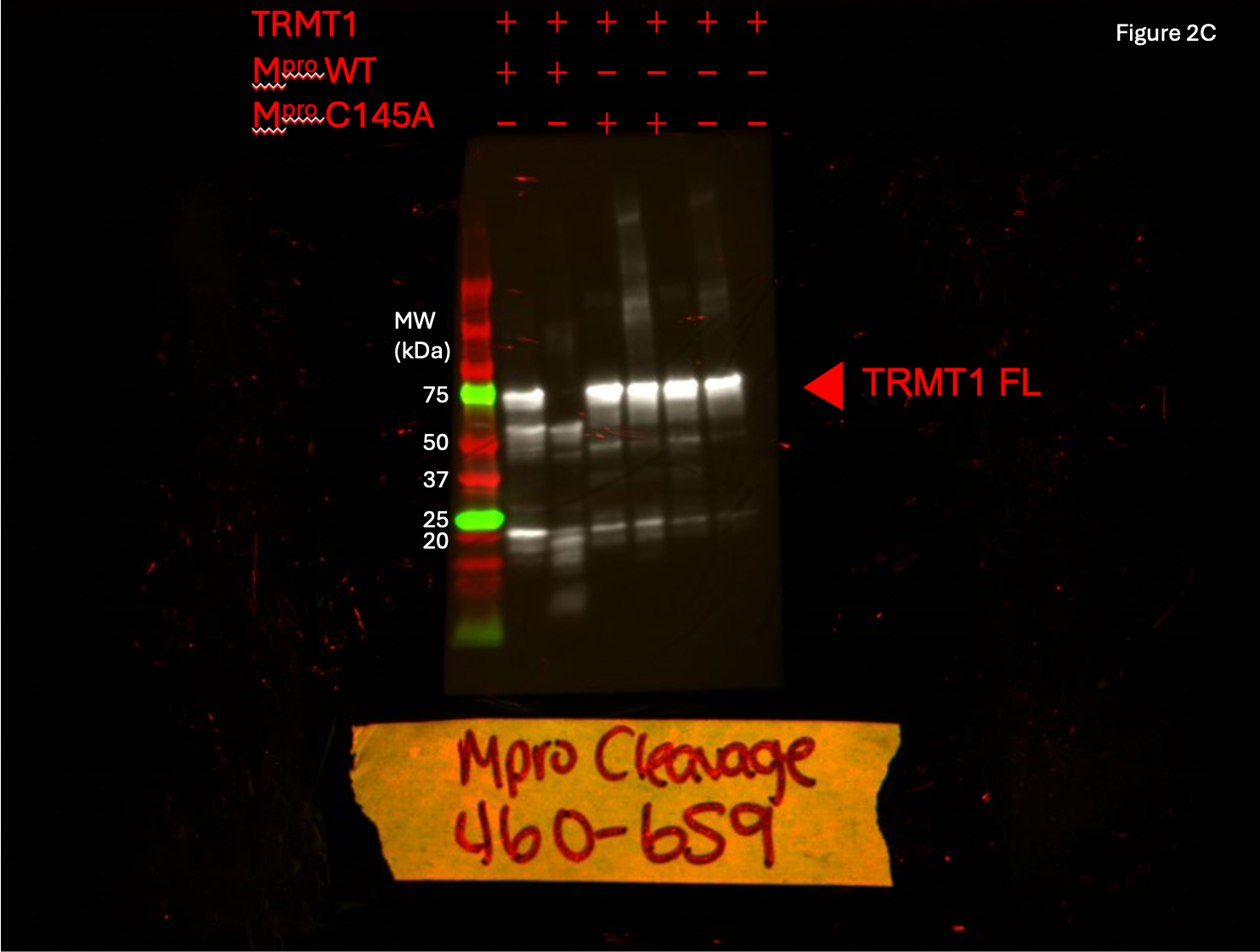

Supplement: Figure 2—source data 3. [file elife-91168-fig2-data3.zip › Figure 2C-annotated-gel.png]

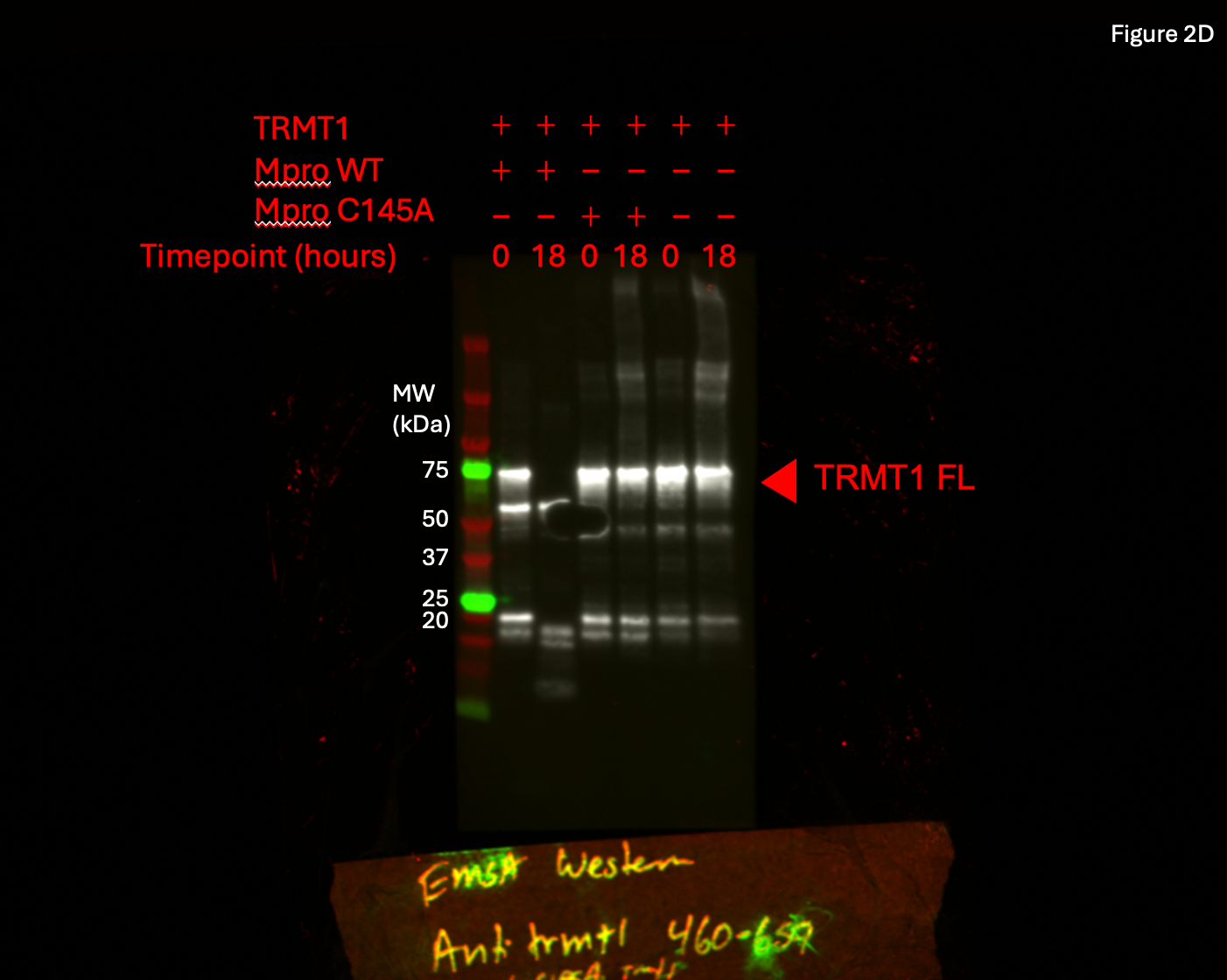

Supplement: Figure 2—source data 3. [file elife-91168-fig2-data3.zip › Figure 2D-annotated-gel.png]

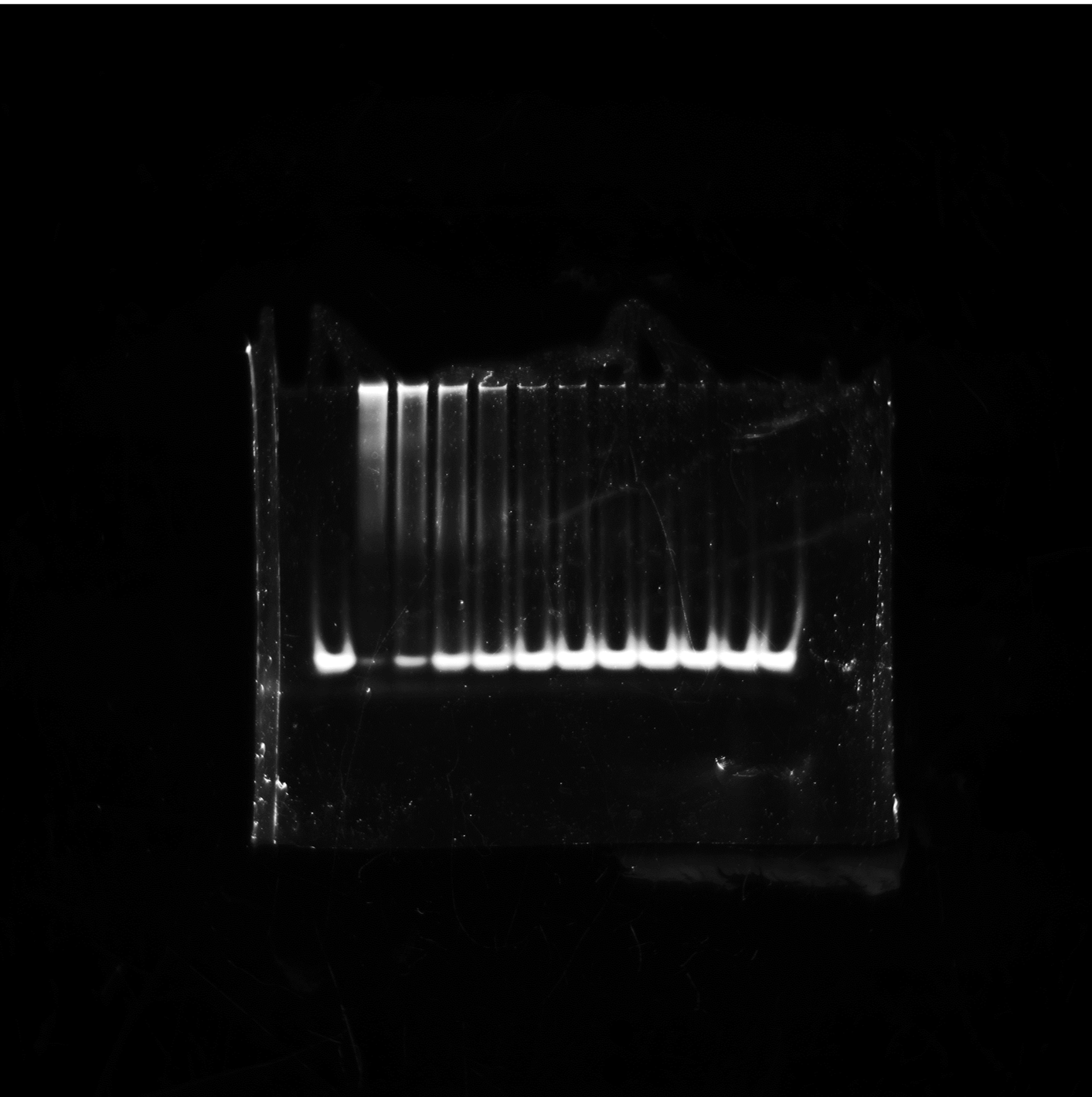

Supplement: Figure 2—figure supplement 1—source data 1. [file elife-91168-fig2-figsupp1-data1.zip › Figure 2-figure supplement 1A-uncropped-gel.png]

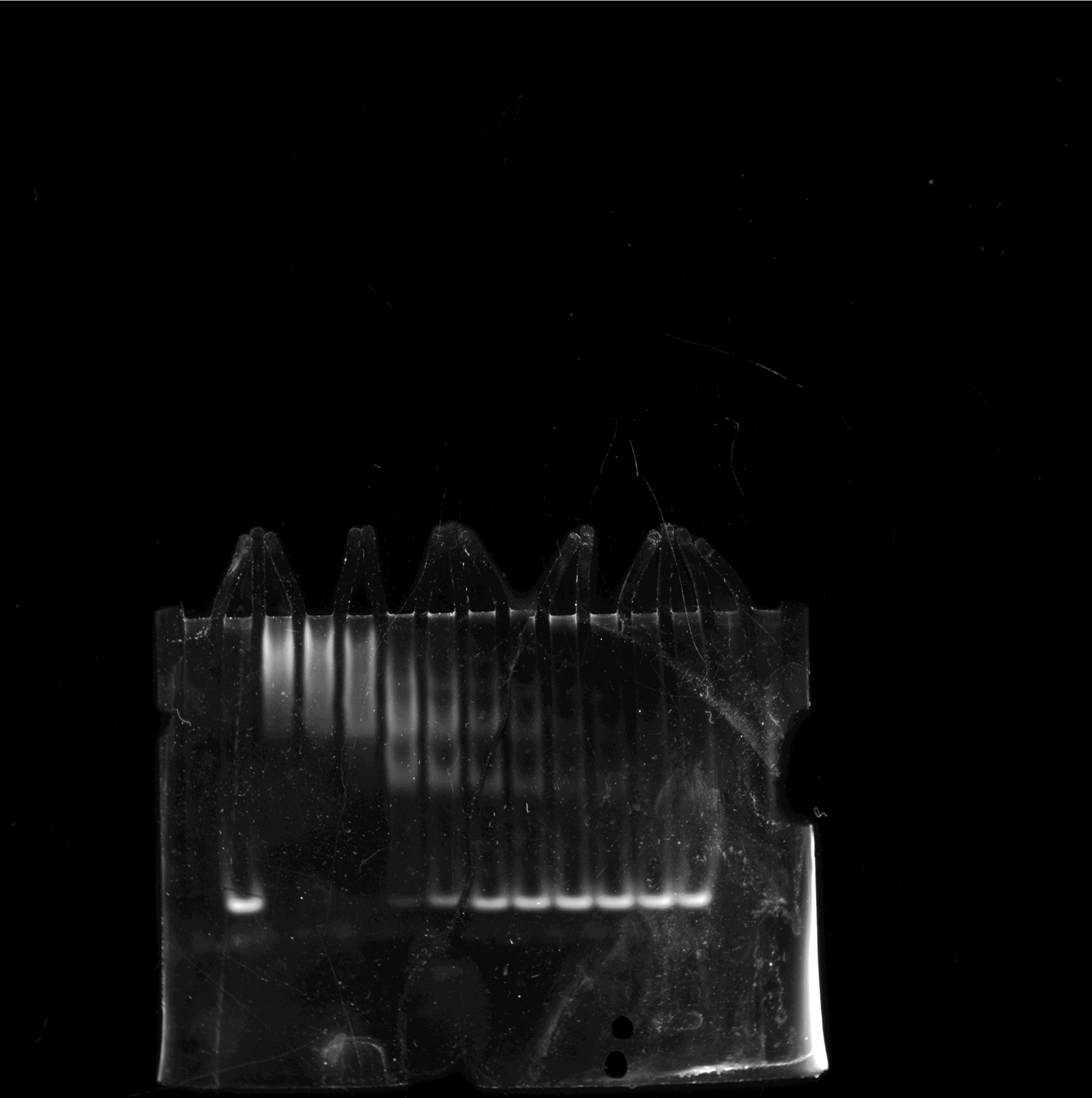

Supplement: Figure 2—figure supplement 1—source data 1. [file elife-91168-fig2-figsupp1-data1.zip › Figure 2-figure supplement 1B-uncropped-gel.png]

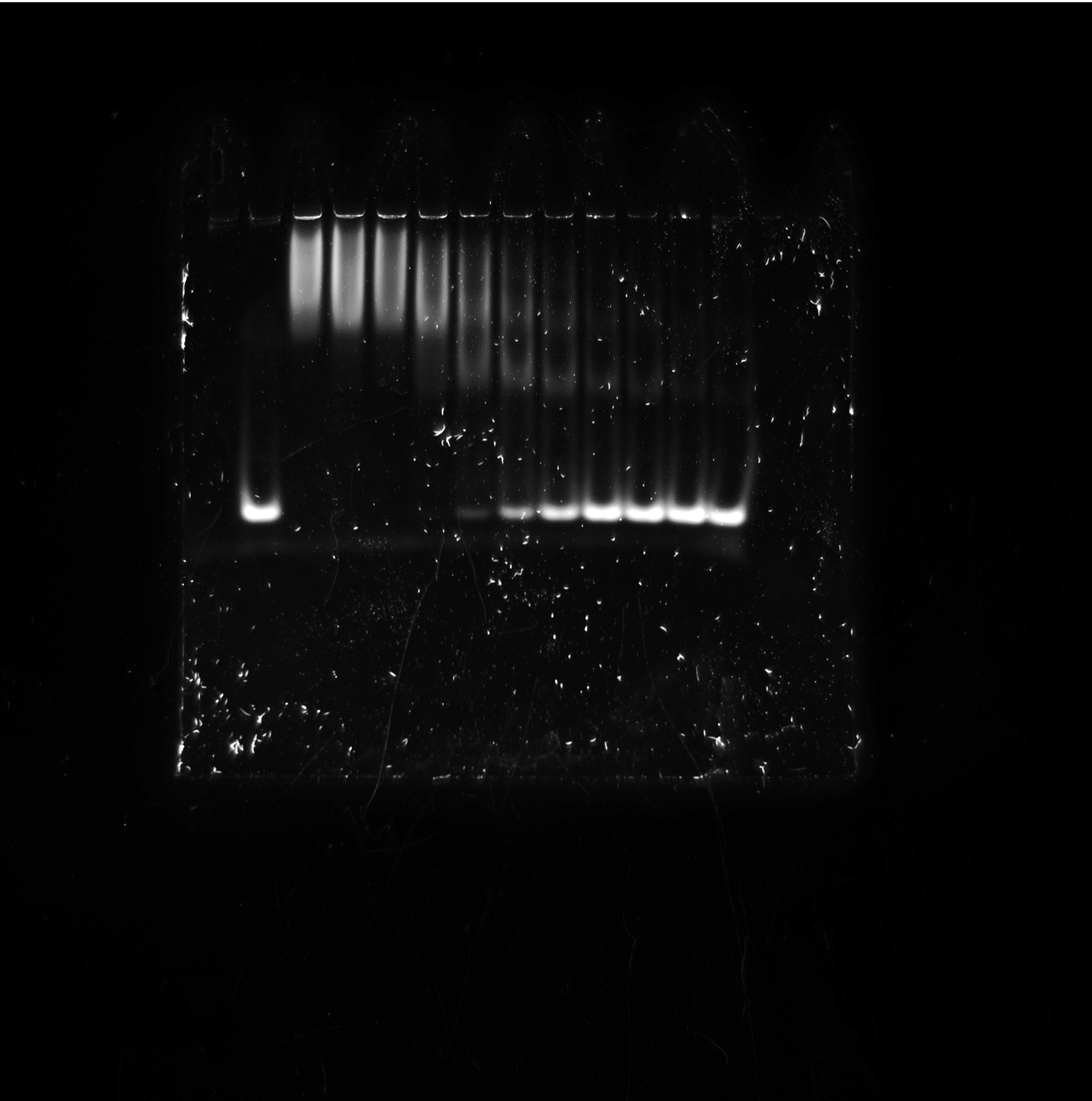

Supplement: Figure 2—figure supplement 1—source data 1. [file elife-91168-fig2-figsupp1-data1.zip › Figure 2-figure supplement 1C-uncropped-gel.png]

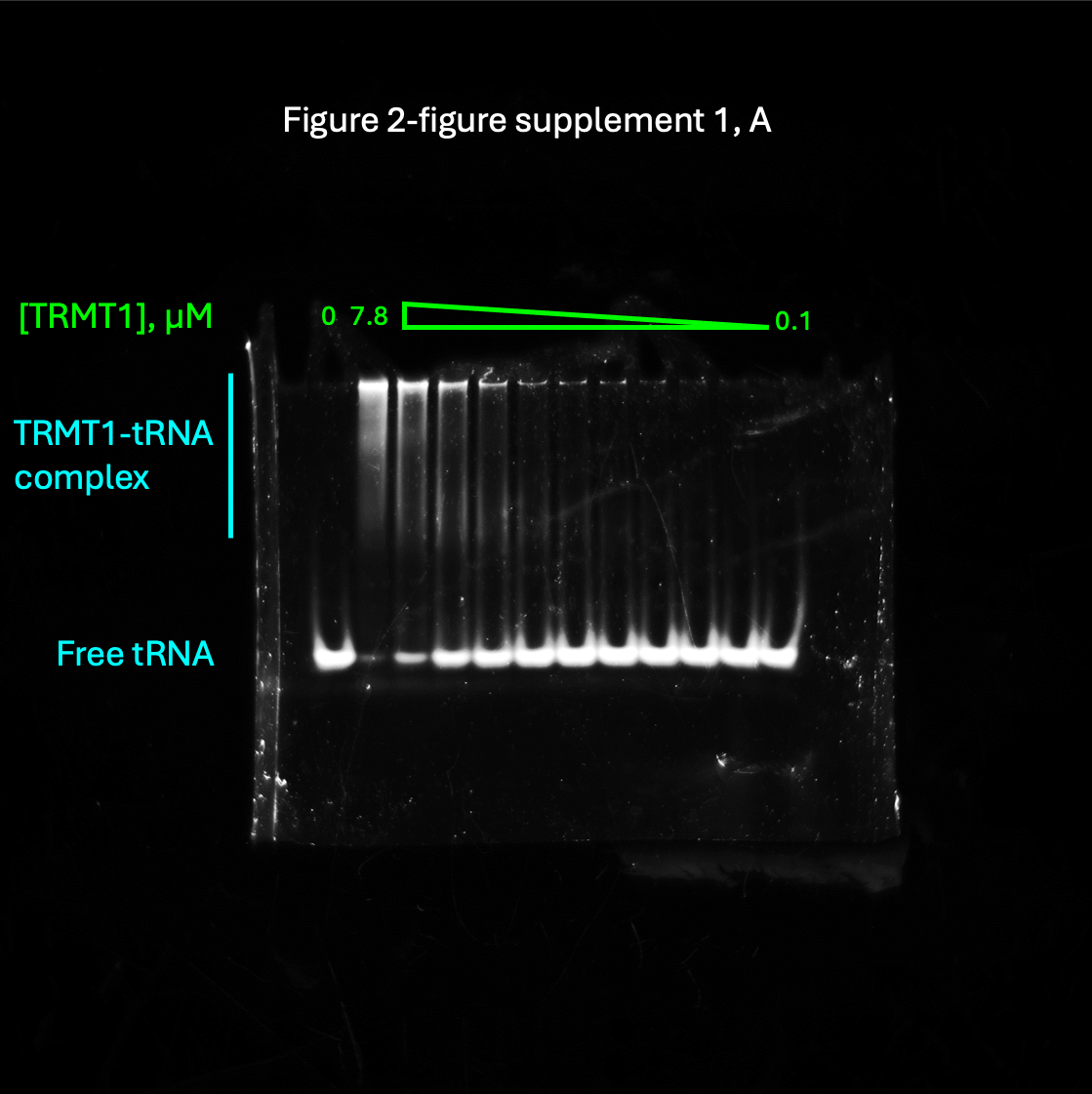

Supplement: Figure 2—figure supplement 1—source data 2. [file elife-91168-fig2-figsupp1-data2.zip › Figure 2-figure supplement 1A-labeled-gel.png]

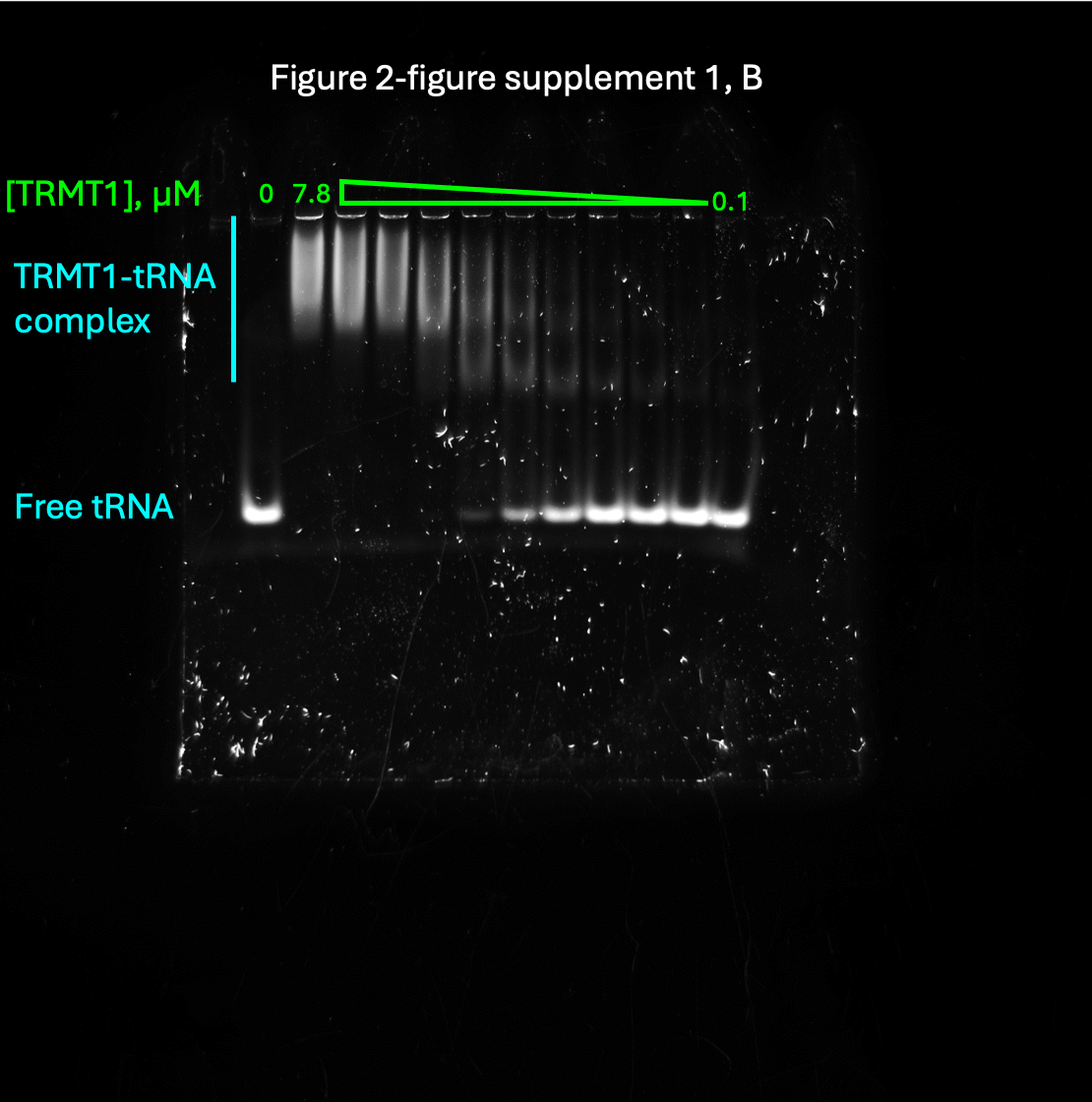

Supplement: Figure 2—figure supplement 1—source data 2. [file elife-91168-fig2-figsupp1-data2.zip › Figure 2-figure supplement 1B-labeled-gel.png]

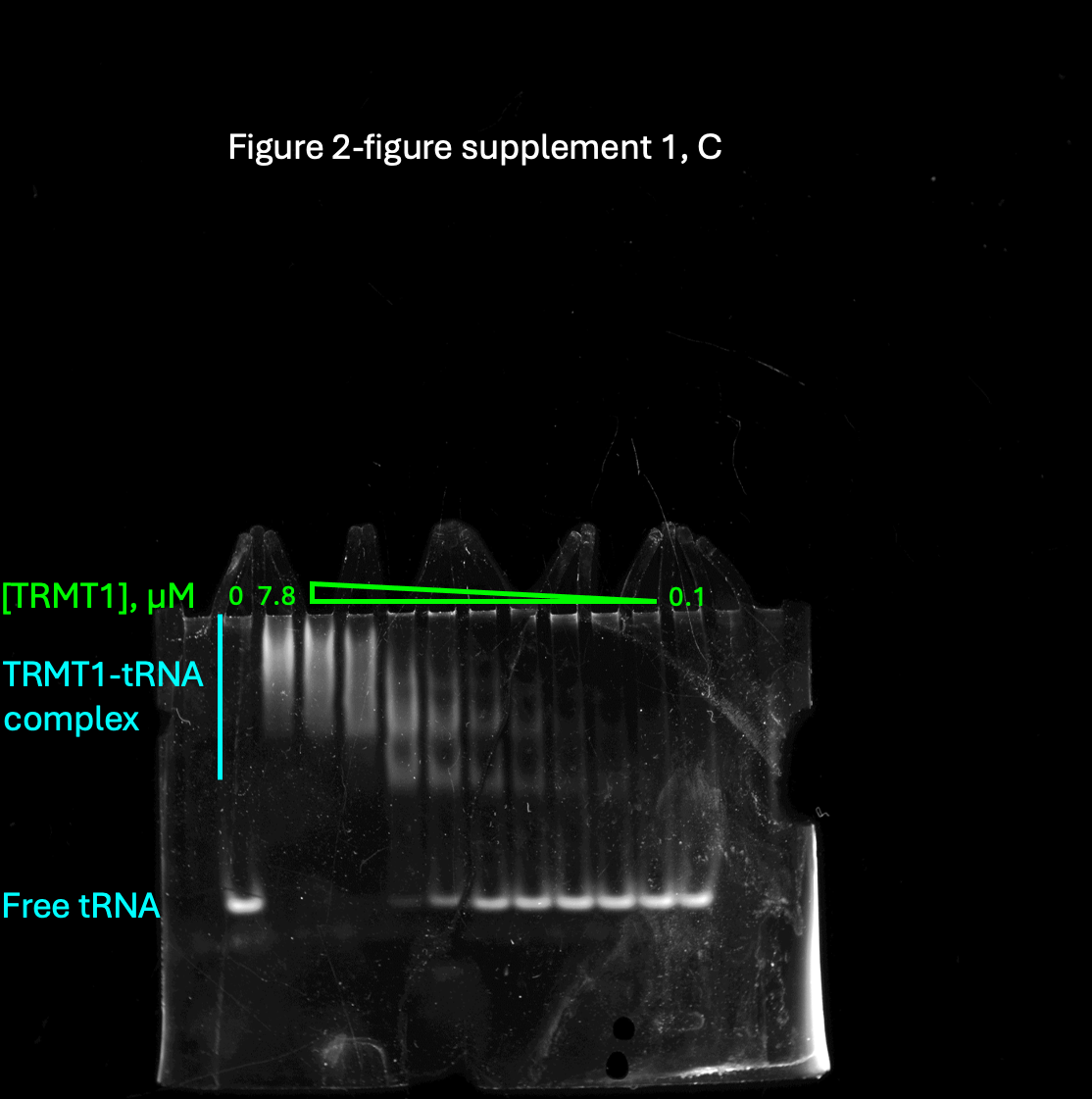

Supplement: Figure 2—figure supplement 1—source data 2. [file elife-91168-fig2-figsupp1-data2.zip › Figure 2-figure supplement 1C-labeled-gel.png]

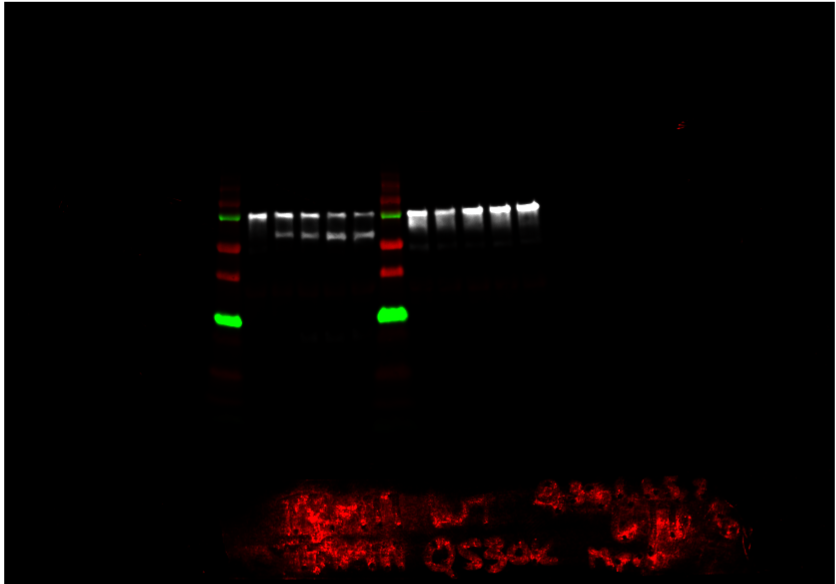

Supplement: Figure 6—source data 2. [file elife-91168-fig6-data2.zip › Figure 6.png]

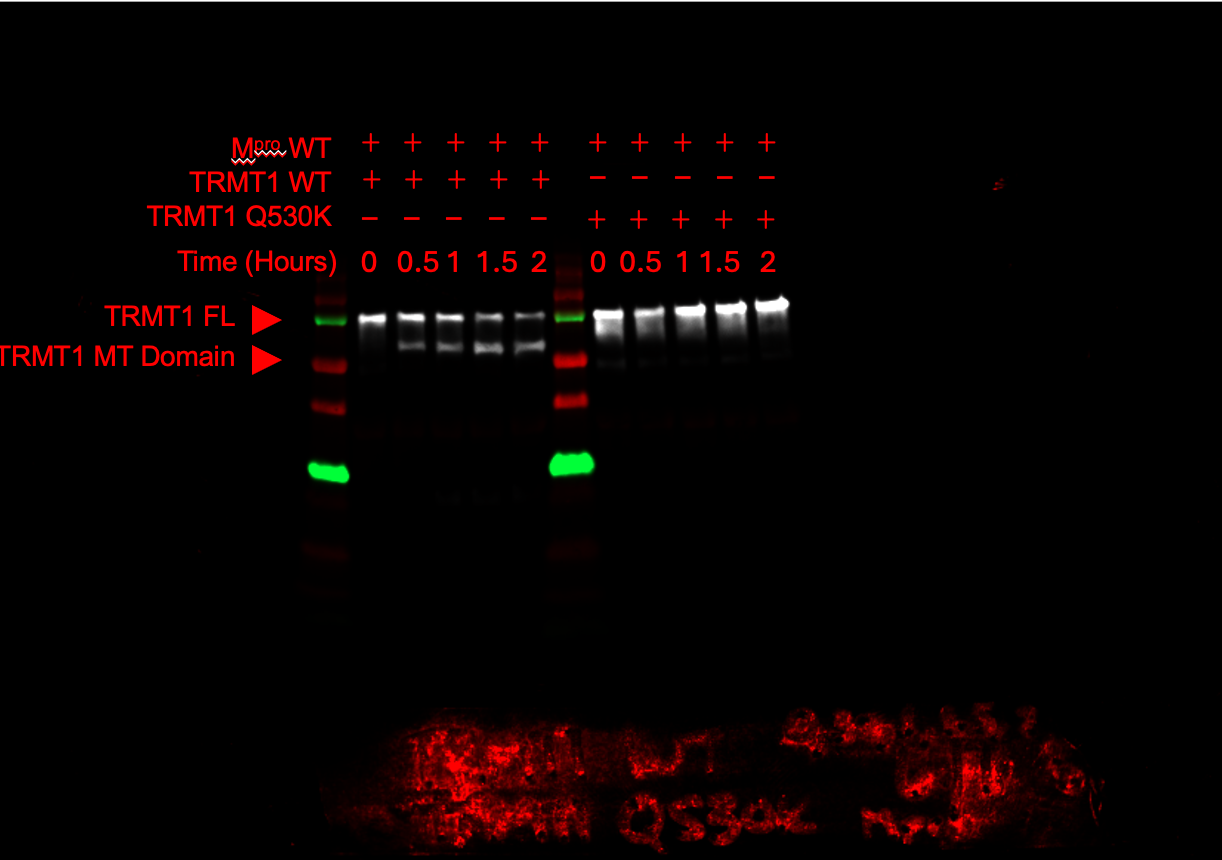

Supplement: Figure 6—source data 3. [file elife-91168-fig6-data3.zip › Figure 6-labeled.png]
