# Supplementary material for: Recognition and cleavage of human tRNA methyltransferase TRMT1 by the SARS-CoV-2 main protease
Source: eLife. 2025 Jan 7;12:RP91168. doi: 10.7554/eLife.91168 (PMC11706605; doi:10.7554/eLife.91168)
Supplement: Figure 3—source data 1. [file elife-91168-fig3-data1.docx]

|  | Crystal structure of SARS-CoV-2 main protease (M^pro^) C145A mutant in complex with peptide from human tRNA methyltransferase TRMT1 (PDB 9DW6) |
| --- | --- |
| **Data collection** |  |
| Space group | P 21 21 21 |
| Cell dimensions |  |
| *a*, *b*, *c* (Å) | 67.79, 100.03, 103.25 |
| α, β, γ (°) | 90.00, 90.00, 90.00 |
| Resolution (Å) | 29.34 – 1.90 (1.97 – 1.90)^a^ |
| *R*_merge_ | 0.21 (1.75) |
| *I* / σ*I* | 9.7 (1.5) |
| *CC*_1/2_ | 99.7 (65.6) |
| Completeness (%) | 99.1 (98.3) |
| Multiplicity | 13.8 (14.0) |
|  |  |
| **Refinement** |  |
| Resolution (Å) | 29.34 – 1.90 |
| No. reflections | 55,515 (5,407) |
| *R* / *R*_free_ | 0.180 / 0.218 |
| No. non-H atoms |  |
| Protein | 4719 |
| Ligand | 15 |
| Water | 356 |
| *B*-factors |  |
| Protein | 32.72 |
| Ligand | 39.75 |
| Water | 42.02 |
| R.m.s. deviations |  |
| Bond lengths (Å) | 0.007 |
| Bond angles (°) | 0.83 |
| Ramachandran plot statistics |  |
| No. favored | 598 (98.17 %) |
| No. allowed | 11 (1.83 %) |
| No. outliers | 0 (0 %) |

Data set was collected from a single crystal. ^a^Values in parentheses are for highest-resolution shell.

**Figure 3–source data 1.** Data and refinement statistics for crystal structure of SARS-CoV-2 main protease (M^pro^) in complex with peptide from human tRNA methyltransferase TRMT1 (PDB 9DW6).
