# Supplementary material for: Recognition and cleavage of human tRNA methyltransferase TRMT1 by the SARS-CoV-2 main protease
Source: eLife. 2025 Jan 7;12:RP91168. doi: 10.7554/eLife.91168 (PMC11706605; doi:10.7554/eLife.91168)
Supplement: Figure 5—source data 1. [file elife-91168-fig5-data1.docx]

| **Substrate** | **M^pro^**  **Mutation** | **[M^pro^]**  **(µM)** | ***k*_cat_**  **(s^-1^)** | ***k*_cat_**  **+/-** | ***K_M_***  **(µM)** | ***K_M_***  **+/-** | ***k*_cat_/*K_M_***  **(µM^-1^s^-1^)** | ***k*_cat_/*K_M_***  **+/-** |
| --- | --- | --- | --- | --- | --- | --- | --- | --- |
| nsp4/5 | WT | 0.05 | 1.05 | 0.05 | 109 | 8 | 0.0097 | 0.0003 |
| nsp4/5 | M49A | 0.05 | 0.41 | 0.03 | 75 | 10 | 0.0055 | 0.0003 |
| nsp4/5 | N142A | 0.05 | 1.06 | 0.07 | 122 | 13 | 0.0087 | 0.0003 |
| nsp4/5 | Q189A | 0.05 | 0.5 | 0.1 | 86 | 29 | 0.0061 | 0.0009 |
| nsp8/9 | WT | 0.05 | 0.019 | 0.002 | 40 | 8 | 0.00050 | 0.00005 |
| TRMT1 | WT | 0.05 | 0.0052 | 0.0002 | 29 | 3 | 0.00020 | 0.00001 |
| TRMT1 | M49A | 0.05 | 0.0064 | 0.0003 | 24 | 3 | 0.00030 | 0.00002 |
| TRMT1 | N142A | 0.05 | 0.0081 | 0.0004 | 31 | 4 | 0.00030 | 0.00002 |
| TRMT1 | Q189A | 0.05 | 0.0062 | 0.0005 | 38 | 7 | 0.00020 | 0.00002 |
| TRMT1(A531S) | WT | 0.05 | 0.0040 | 0.0007 | 22 | 8 | 0.00020 | 0.00004 |

**Figure 5–source data 1.**  Michaelis–Menten kinetics determined for different fluorogenic peptide substrates cleaved by M^pro^ wild-type (WT) and M^pro^ mutants (M49A, N142, Q189A). Individual kinetic measurements were carried out in triplicate and the +/- columns denote the standard errors on each parameter derived from non-linear least squares regression fits.
